# Editorial: The role of primary and community care in rehabilitation

**DOI:** 10.3389/fresc.2023.1235049

**Published:** 2023-07-12

**Authors:** Armin Gemperli, Stefan Essig

**Affiliations:** ^1^ Health Services Research, Swiss Paraplegic Research, Nottwil, Switzerland; ^2^ Center for Primary and Community Care, University of Lucerne, Lucerne, Switzerland

**Keywords:** primary care, community care, rehabilitation, care fragmentation, care coordination, functioning, ICF (international classification of functioning), family medicine

**Editorial on the Research Topic** The role of primary and community care in rehabilitation

## Background

Rehabilitation aims at improving people's functional abilities, restoring or increasing independence and promoting optimal recovery (1). It has been emphasized that rehabilitation can be provided by a variety of health professionals, including primary care workers (2). The WHO in its “Rehabilitation 2030: A call for action” aims to ensure that rehabilitation is recognized as an essential health strategy and integrated into health systems (3). It emphasizes the importance of person-centered and community-based rehabilitation. In the report of the second Rehabilitation 2030 meeting, the WHO presents two examples of integrating rehabilitation into primary care from Eswatini and the Philippines (4). However, the potential of rehabilitation in primary and community care has not yet been fully realized.

Primary care refers to the first point of contact for individuals seeking healthcare services, typically provided by healthcare professionals such as general practitioners, family physicians, or pediatricians. It encompasses comprehensive, continuous, and coordinated healthcare services that are aimed at addressing a wide range of health needs, promoting preventive care, managing common illnesses, and providing ongoing care for chronic conditions. The importance of primary care is recognized in the Astana Declaration, a landmark document adopted at the Global Conference on Primary Health Care held in Astana, Kazakhstan in 2018 (5). The Astana Declaration reaffirms the commitment of countries to strengthen primary care as the foundation for achieving universal health coverage, health equity, addressing social determinants of health, and delivering person-centered care.

Both rehabilitation and primary care advocates see demographic and epidemiologic trends as necessitating a shift in health system strategies, with a greater focus on their respective areas. This is not a contradiction if one considers better integrating both strategies in future healthcare systems. The field of rehabilitation has been described as “highly fragmented” with many different rehabilitation professionals and subspecialties (6). This is where primary care providers can excel in their core discipline: coordinating care to ensure that individuals receive comprehensive and integrated care, including rehabilitation services. They can ensure effective referral and follow-up. Functional collaboration between general practitioners and rehabilitation professionals is required for primary care to function as an effective gateway to rehabilitation services, including well-defined roles and patient pathways. While such care coordination is implicitly assumed, it has rarely been conceptualized (7), nor have many different collaboration models and their effectiveness been illustrated.

## Research outlook

Under this journal's research topic “The role of primary and community care in rehabilitation”, we called for research on overcoming the fragmentation of care. The papers illustrate the relatively advanced integration of rehabilitation services into primary care, namely in Canada and Denmark, where further evidence was presented on improving quality initiatives, team communication or patient adherence and dropout.

Cardiac rehabilitation is provided in the primary care setting in Denmark. The setting allows for interprofessional care including nurses, physiotherapists or dieticians. Identified problems are related to patient adherence and dropout (Raven et al., 2022a and Raven et al., 2022b). The primary care setting has been identified as more accessible than the hospital setting, where the primary care physicians have established long-term relationships with their patients, gaining a deeper understanding of their health histories, preferences, and social contexts. Still, it remains unclear who oversees treatment and takes responsibility for following up on rehabilitation goals. Is it still the hospital, of which the primary care center is only an external ward, or does the primary care provider assume this role? In the former case, care may follow more quickly from the initial hospitalization, but the new environment also challenges it. In the second case, additional efforts are needed to align the goals of the different settings while simultaneously providing care closer to the patient's social and cultural environment. The authors conclude that it is crucial to address the broader perspective of patients to promote adherence. While the closed social setting may be less intimidating to patients, it is also where they feel less obligated to adhere. In a patient-centered approach with shared decision-making, non-adherence and dropout must be accepted if the patient is fully informed. Family physicians can provide a long-term relationship and a source of trust that allows functioning goals to be pursued even in situations where the patient is not adhering to the optimal treatment regimen. A factor associated with dropout from cardiac rehabilitation in primary care was the long travel time to the cardiac rehabilitation center. This suggests that integrating rehabilitation into primary care and general practice is not yet as detailed as it should be.

Another research project examined the quality of collaboration among rehabilitation team members in primary care in Canada (Wener et al., 2022). The Interprofessional Collaborative Relationship-Building Model (ICRB) was proposed as a tool for understanding the stages of development of building interprofessional team relationships. The two central processes of the model are communication strategies and patient-centeredness. The research examines the applicability of this model in primary care, specifically the development of collaborative relationships between occupational and physical therapists and the core primary care team. Specifically in Canada, a growing number of occupational and physical therapists have been integrated into a primary care team that typically includes physicians, nurses, social workers, and dieticians. The research emphasizes that patient-centeredness and quality face-to-face communication are building blocks for collaborative relationship building. Although the ICRB model appears to be a good starting point for developing collaborative relationships, the researchers also emphasize that such interprofessional teams in primary care need to develop over time.

To improve the coordination and quality of rehabilitation in primary care, the mobilization of registry data has been proposed (Krysa et al., 2023). Primary care has been identified as an integral part of coordinating care between patients, different rehabilitation providers and community care partners. This care integration can be facilitated by clinical data, which can come from various sources, including clinical registries. Central to this effort is the availability of information about an individual's functional status, including their strengths, limitations, and needs in performing activities of daily living. Functioning data are essential for determining the impact of a health condition or disability on a patient's daily life, for making accurate diagnoses, for guiding the development of appropriate treatment plans, including support services and assistive devices, and for subsequent monitoring. Functioning information goes beyond medical outcomes to capture the impact of health conditions on a person's daily functioning, social participation, and overall well-being, enabling personalized care planning. However, lack of organizational support, limited resources, lack of theoretical frameworks for evaluating existing data, data and privacy restrictions, and inadequate or siloed information technology structures are barriers to supporting health information exchange. While the International Classification of Functioning, Disability and Health (ICF) has been promoted as ideal for quality assessment in rehabilitation, it has also been identified as time-consuming and potentially disruptive to sampling during episodes of care (8). Therefore, the inclusion of clinical registry data is complementary, with the research presented providing practical strategies and priorities for using registry data.

The fifth study of our collection deals with another topic. In Bangladesh, studies show that patient satisfaction with healthcare services is low in public primary care facilities and public hospitals (Begum et al. 2022). Satisfaction will be another important outcome measure if we want to improve patient pathways across different healthcare settings.

## Summary

In summary, the research presented provides practical insights into different rehabilitation settings in primary care and offers insights into how to improve quality measurement, team communication, patient adherence and dropout. A common theme is the focus on patient needs as a guiding principle. These studies are also an illustration of the wide range of implementation of rehabilitation in primary care. This area is still underdeveloped, with different health systems struggling to integrate such services. A key issue is the empowerment of primary care physicians to manage rehabilitation in primary care, which requires training at the undergraduate or postgraduate level. Depending on the complexity of the health condition, a collaborative or shared care model with secondary or even tertiary care specialists must be considered. Hopefully, there will be more illustrations, implementation frameworks and best practice models to learn from and progressively achieve better health by providing rehabilitation services closer to patients’ needs in primary care and community settings.
